# The Anti-Inflammatory Drug Leflunomide Is an Agonist of the Aryl Hydrocarbon Receptor

**DOI:** 10.1371/journal.pone.0013128

**Published:** 2010-10-01

**Authors:** Edmond F. O'Donnell, Katerine S. Saili, Daniel C. Koch, Prasad R. Kopparapu, David Farrer, William H. Bisson, Lijoy K. Mathew, Sumitra Sengupta, Nancy I. Kerkvliet, Robert L. Tanguay, Siva Kumar Kolluri

**Affiliations:** 1 Cancer Research Laboratory, Environmental Health Sciences Center, Oregon State University, Corvallis, Oregon, United States of America; 2 Department of Environmental and Molecular Toxicology, Environmental Health Sciences Center, Oregon State University, Corvallis, Oregon, United States of America; 3 Pharmaceutical Biochemistry Group, School of Pharmaceutical Sciences, University of Geneva, Geneva, Switzerland; University Paris Diderot-Paris 7, France

## Abstract

**Background:**

The aryl hydrocarbon receptor (AhR) is a ligand-activated transcription factor that mediates the toxicity and biological activity of dioxins and related chemicals. The AhR influences a variety of processes involved in cellular growth and differentiation, and recent studies have suggested that the AhR is a potential target for immune-mediated diseases.

**Methodology/Principal Findings:**

During a screen for molecules that activate the AhR, leflunomide, an immunomodulatory drug presently used in the clinic for the treatment of rheumatoid arthritis, was identified as an AhR agonist. We aimed to determine whether any biological activity of leflunomide could be attributed to a previously unappreciated interaction with the AhR. The currently established mechanism of action of leflunomide involves its metabolism to A771726, possibly by cytochrome P450 enzymes, followed by inhibition of *de novo* pyrimidine biosynthesis by A771726. Our results demonstrate that leflunomide, but not its metabolite A771726, caused nuclear translocation of AhR into the nucleus and increased expression of AhR-responsive reporter genes and endogenous AhR target genes in an AhR-dependent manner. *In silico* Molecular Docking studies employing AhR ligand binding domain revealed favorable binding energy for leflunomide, but not for A771726. Further, leflunomide, but not A771726, inhibited *in vivo* epimorphic regeneration in a zebrafish model of tissue regeneration in an AhR-dependent manner. However, suppression of lymphocyte proliferation by leflunomide or A771726 was not dependent on AhR.

**Conclusions:**

These data reveal that leflunomide, an anti-inflammatory drug, is an agonist of the AhR. Our findings link AhR activation by leflunomide to inhibition of fin regeneration in zebrafish. Identification of alternative AhR agonists is a critical step in evaluating the AhR as a therapeutic target for the treatment of immune disorders.

## Introduction

The aryl hydrocarbon receptor (AhR) is a member of the Per-AhR/Arnt-Sim (PAS) family of proteins. The AhR is a cytosolic transcription factor that, in its latent unliganded state, forms complexes with HSP90 and XAP2.[1] Upon ligand binding, the AhR translocates to the nucleus, where it complexes with its heterodimerization partner, the AhR Nuclear Translocator (Arnt), to modulate expression of AhR target genes containing functional xenobiotic response elements (XREs).[1] Activation of AhR by 2,3,7,8-tetrachlorodibenzo-p-dioxin (TCDD) is associated with a number of adverse effects in animals including tumor promotion and immune suppression.[2] Studies have shown that the AhR, upon activation by TCDD, inhibits cellular proliferation by inducing expression of cell cycle inhibitor p27^Kip1^.[3] Interaction of the AhR with retinoblastoma protein has also been reported. [4], [5], [6] Further, the AhR has been shown to modulate cell cycle progression and cellular differentiation independent of TCDD.[7] In addition, the AhR can also modulate tissue regeneration pathways *in vivo*.[8], [9] The AhR can induce mitogen-activated protein kinases as well as modulate function of tyrosine kinases.[10], [11] Despite the negative physiological effects associated with TCDD activation of AhR *in vivo*, recent studies on the AhR suggest that this receptor may play a role in the control of tumor progression in the absence of exogenous compounds and further, that modulators of the AhR may be useful as therapeutics for immune-mediated diseases and cancer.[12], [13], [14], [15], [16]

In the present study, we conducted a screen of clinically used compounds in order to identify novel AhR ligands and identified the anti-rheumatoid arthritis drug leflunomide as a putative AhR activator. Consistent with this result of our screen, Hu *et al* previously reported leflunomide as an AhR activator during a study evaluating the usefulness of CYP1A1 as a biomarker of AhR activation.[17] Leflunomide (Arava®) is an immunomodulatory drug whose primary mechanism of action is attributed to its metabolite A771726 via inhibition of dihydroorotate dehydrogenase, which in turn disrupts pyrimidine biosynthesis and ultimately inhibits T and B cell proliferation.[18] Potent suppression of the immune response by TCDD is a well-known AhR-dependent phenomenon.[15], [19], [20] Because leflunomide is both an agonist of the AhR and a known immunosuppressive agent, we aimed to determine if the biological activity of leflunomide could be attributed to a previously unappreciated activation of the AhR. In addition, given that one of the most well known roles of the AhR is activation of drug metabolizing enzymes upon binding to xenobiotics,[1] we also investigated leflunomide's ability to regulate several genes involved in phase 1 and phase 2 metabolism in an AhR-dependent manner. This investigation included CYP1A2, which has been shown previously to facilitate leflunomide conversion to A77176.[21]. Lastly, we investigated *in vivo* activation of the AhR and initiation of non-immune related AhR responses by leflunomide.

## Materials and Methods

### Cell Culture

Mouse WT Hepa1c1c7 hepatoma cells, mouse Hepa1 B13NBii1 (C4) cells,[22] mouse vT{2} cells,[23] mouse Hil1.1c2 cells,[24] and human HepG2 hepatoma cells were cultured in DMEM with L-glutamine (Mediatech Inc., Manassas, VA) supplemented with 10% FBS (Tissue Culture Biologicals, Tulare, CA) in a humidified 5% CO_2_ atmosphere. Mouse WT Hep1c1c7 cells are hereafter referred to as WT Hepa1, while Hil1.1c2 cells are hereafter referred to as Hepa1.1 cells. Mouse Hepa1 C4 and VT{2} cells are derivatives of the WT Hepa1 cell line and were purchased from ATCC. Mouse Hepa1 C4 cells lack functional Arnt activity due to a point mutation (GLY326ASP),[25] while vT{2} cells are C4 cells engineered to stably express a full length Arnt cDNA.[23] Mouse Hepa1.1 cells,[24] were kindly provided by Dr. M.S. Dension (University of California, Davis, CA, USA). All cell lines were cultured with 100 U/mL penicillin and 100 mg/mL streptomycin (Mediatech Inc., Manassas, VA), and were typically passaged every three days at a dilution of 1∶5. Mouse splenocytes used in *ex vivo* experiments were cultured in RPMI 1640 media (Invitrogen, Carlsbad, CA) supplemented with 10% FBS without antibiotics.

### Chemicals and Reagents

Leflunomide and A771726 were purchased from Sigma (St Louis, MO) and Alexis Biochemicals (Plymouth Meeting, PA), respectively, and dissolved in DMSO. Fluvoxamine Maleate, a chemical inhibitor of CYP1A1 and CYP1A2, was purchased from Tocris Biosciences (Ellisville, MO) and dissolved in DMSO. All other chemicals, the chemical library, and reagents were purchased from Sigma (St Louis, MO) unless otherwise noted.

### Reporter Gene Constructs

The following constructs were used for reporter gene assays. Hepa1.1 cells stably express pGudLuc1.1, an expression vector with a 484-base pair fragment of the promoter region of mouse *cyp1a1* that contains four functional XRE sequences inserted directly upstream of the mouse mammary tumor virus (MMTV) viral promoter and firefly luciferase gene.[24] For transient transfection assays in WT Hepa1, C4, vT{2}, and HepG2 cells, the XRE-MMTV-Luc expression vector was used. XRE-MMTV-Luc, hereafter referred to as XRE-Luc, contains a synthetic XRE oligonucleotide upstream of the MMTV viral promoter.[26]. The β-galactosidase expression vector, which expresses the β-galactosidase gene under control of a minimal CMV promoter (pCH 110; Pharmacia), was used for normalization of luciferase activity as described previously.[27]. PCDNA3.0 (Invitrogen) was used as carrier DNA for transfection normalization purposes. Reconstitution of Arnt activity in C4 cells was achieved by transfecting cells with an expression vector for Arnt.[28]

### Reporter Gene Assays

Hepa1.1 cells were plated at a density of 1×10^4^ cells/well in 100 µL of cell culture media in 96 well plates and grown overnight. The following day, cells were treated for four hours with vehicle (DMSO), leflunomide, or A771726; the total concentration of DMSO did not exceed 0.1% v/v. Following incubation with the compounds, the media was removed and cells were harvested with 100 µL passive lysis buffer (Promega) for 15 min with mild orbital shaking. Next, 75 µL of the resulting lysates were collected and transferred to opaque 96 well plates, where they were assayed well-by-well for luciferase activity by injection of luciferase assay substrate (Promega) with a 2 sec mixing time and 15 sec integration period on a Tropix TR717 microplate luminometer. Data were expressed as fold inductions relative to vehicle (DMSO) treated cells.

For transient transfections, Hepa1, vT{2}, C4, and HepG2 cells were plated at a density of 0.75×10^5^ cells/well in 24 well plates and grown overnight. The following day the cells were transfected with 600 ng of the XRE-Luc expression vector, 100 ng β-galactosidase expression vector, and 300 ng PCDNA3.0 as carrier DNA using Lipofectamine 2000 (Invitrogen, CA) according to the manufacturer's recommended protocol. Co-transfection with the β-galactosidase expression vector was for normalization purposes as described below. Pertaining specifically to reconstitution of Arnt activity in C4 cells, which lack functional Arnt activity, cells were co-transfected with 300 ng Arnt expression vector or with PCDNA3.0 carrier DNA, thereby keeping the total amount of DNA transfected equal among experiments.

Approximately 18 hours after transfection the media was removed and the cells were treated with DMSO, leflunomide, or A771726 for an additional 18 hours; the total concentration of DMSO in experiments did not exceed 0.1% v/v. After incubation with the compounds, cells were lysed essentially as described above for Hepa1.1 experiments, except that the total lysate volume was increased to 150 µL, the amount of lysate assayed in the luminometer was increased to 100 µL, and 10–25 µL of lysate was used for mouse and human β-galactosidase assays for transfection normalization, respectively.[29], [30], [31], [32] Briefly, β-galactosidase activity was determined by incubating a portion of the cell lysates for approximately 20 minutes with 100 µL β-galactosidase reaction buffer per well (100 mM sodium phosphate buffer pH 7.3, 1.25 mM MgCl_2_, 62.5 mM 2-Mercaptoethanol, and 1.1 mg/mL O-nitrophenyl-beta-D-galactopyranoside) at 37°C, and reading the absorbance at 405 nm in a spectromax 96-well plate reader. To normalize the data in transient transfection experiments, raw luciferase values were divided by their respective β-galactosidase activity. Values are presented as fold inductions relative to vehicle treated cells.

### Chemical Inhibition of CYP1A2

Reporter assays utilizing chemical inhibition of CYP1A2 were performed as described above for the Hepa1.1 reporter assay, with some modifications. Briefly, prior to treatment with vehicle (DMSO) or leflunomide, cells were pre-treated for 3 hours with fluvoxamine at a final concentration of 10 µM. Cells were then treated for 4 hours with vehicle or leflunomide and luciferase activity was measured as described above.

### Semi-quantitative Polymerase Chain Reaction

For analysis of AhR target gene induction, Hepa1, C4, VT{2}, or HepG2 cells were plated in 6 well dishes and grown overnight such that they were 50% confluent at the time of treatment. Cells were then treated with vehicle (DMSO) and varying levels of either leflunomide or A771726 for approximately 18 hours, at which time they were harvested using the RNeasy kit (Qiagen) according to the manufacture's recommended protocol. The concentration of RNA lysates were quantified with a nanodrop spectrophotometer and frozen at −20°C until needed. Reverse Transcriptase PCR analysis was performed using the Superscript RT III Kit with random hexamers and a 1 µg RNA Input (Invitrogen, CA). Semi-quantitative Real Time PCR was performed following cDNA synthesis exactly as previously described.[33] Primers were designed to amplify Cytochrome P450 1A1 (CYP1A1), Cytochrome P450 1A2 (CYP1A2), UDP glucuronosyltransferase 1A1 (UGT1A1), NAD(P)H dehydrogenase [quinone 1] (NQO1), and Glyceraldehyde 3-phosphate dehydrogenase (GAPDH), which was used as a control for equal loading. The sequences, accession numbers, and species specificity for the primers used in this study are shown in Table 1. Aliquots of individual PCR reactions at non-saturating PCR conditions were removed at various cycle numbers (indicated in figures) and analyzed by agarose gel electrophoresis on 2.0% TAE gels. Gels were visualized with ethidium bromide using a GeneGenius digital imaging system (Syngene).

**Table 1:** Primer sequences for RT-PCR experiments.

Gene	Accession #	Species	FP (5′-3′)	RP (5′-3′)
**GAPDH**	NM_002046.3	**H/M**	ACCACAGTCCATGCCATCAC 1	TCCACCACCCTGTTGCTGTA1
**CYP1A1**	NM_009992.3	**M**	CTGGTAACCAACCCTAGG 1	CAGGAAGAGAAAGACCTCC 1
**CYP1A2**	NM_009993.3	**M**	TGACCGTCCCCAGCTGCC	GTGGCCATGCCTGGACGTG
**NQO1**	NM_008706.5	**M**	GCCATTCTGAAAGGCTGG 1	CGTTTCTTCCATCCTTCCAG 1
**UGT1A1**	NM_201645.2	**M**	GAACGTGCTCCTGGCCGTG	AGCGCCACAGGACCGTCTG
**CYP1A1**	NM_000499.3	**H**	GTCCCCTTCACCATCCC	CAGGAAGAGAAAGACCTCC
**CYP1A2**	NM_000761.3	**H**	GCCTAGAGCCAGCGGCAACC	GCCATCGGCGGTGAGGAACC
**NQO1**	NM_000903.2	**H**	TCCACCTCAAACGGGCCGG	ACCACTGCAGGGGGAACTGG
**UGT1A1**	NM_000463.2	**H**	TGGCTGAGCATGCTTGGGGC	GCCACGATGGGGCTGCAAGG

**H: Human M: Mouse, FP: Forward Primer, RP: Reverse Primer.**

**^1^** **Sequences described previously.** [**33**]

### Immunofluorescence

WT Hepa1 cells (1×10^4^ cells/well) were plated in 8-well chamber slides and grown overnight. The following day, cells were treated with the indicated compounds for either 1 or 3 hours. At the end of the treatment, cells were processed for immunofluorescence analysis as described previously.[29], [30], [31], [32], [33] Briefly, cells were fixed with 3.7% paraformaldehyde, followed by a 10-minute incubation with 0.1% Triton X-100. After fixing, cells were blocked (1% w/v BSA Fraction V) for 1 hour at room temperature (RT). Primary staining was then performed using an AhR antibody (Biomol, Plymouth Meeting, PA) diluted 1∶800 dilution in blocking medium for 3 hours at RT. After extensive washing with PBS, cells were stained with a FITC conjugated goat-anti-rabbit secondary antibody (Southern Biotech, Birmingham, AL) at a dilution of 1∶600 for 1 hour at RT. Slides were then washed with PBS three times and coverslippped with Pro-Fade staining reagent with DAPI (Molecular Probes, Invitrogen). Cells were imaged with a Zeiss Axiovert 200 inverted microscope equipped with a camera and Metamorph image capture software.

### Molecular Docking Studies

Leflunomide and its metabolite A771726 were docked into a mouse and human AhR ligand binding domain (LBD) homology model using ICM software as described previously, and the binding energy was subsequently calculated.[33]

### Zebrafish Care

AB strain (wildtype) embryos were obtained from the Sinnhuber Aquatic Research Laboratory in the Aquatic Biomedical Models Facility Core of the Environmental Health Sciences Center, Oregon State University. Zebrafish were raised using standard protocols in compliance with Oregon State University animal care and use protocols. Embryos were incubated at 28°C for all experiments.

### Zebrafish Fin Regeneration Assay

The fin regeneration assays were performed as described previously.[9] Briefly, 48 hours post fertilization (hpf) embryos were dechorionated, anesthetized with tricaine (tricaine methanesulfonate; MS-222), placed on a 1% agarose plate, and the primordial caudal fin was amputated just posterior to the notochord using a surgical blade. Embryos were allowed to recover in embryo water approximately 10 minutes followed by a 3-day exposure to vehicle, leflunomide (25 µM), or A771726 (10 µM) diluted in embryo water. Three days post amputation (dpa) embryos were evaluated for mortality, abnormal development, and fin regeneration. Photographs were collected by placing embryos on agarose plates and using a macro camera (Nikon Coolpix 5000) attached to a stereomicroscope (Nikon SMZ1500).

### Zebrafish Morpholino Gene Knockdowns

Antisense repression of AhR2 was performed using a translation start-site targeting morpholino oligonucluotide (MO) (Gene Tools, Philomath, OR) as described previously.[9] All MOs were diluted to 3 mM in 1× Danieau's solution (58 mM NaCl, 0.7 mM KCl, 0.4 mM MgSO4, 0.6 mM Ca(NO3)2, 5 mM HEPES, pH 7.6) as described previously.[34] Next, 2 nL of the AhR2 or standard control MO (Gene Tools) solutions were microinjected into 1–2 cell stage embryos. A fluorescein tag at the 3′ end of the MOs was used to evaluate microinjection efficiency at 24 hpf. Six hpf MO-injected embryos were exposed to vehicle, leflunomide (10 µM), or A771726 (10 µM) and incubated for 3 days followed by toxicity evaluations and immunohistochemistry. A total of 48 hpf embryos were dechorionated and either directly exposed to DMSO (vehicle), leflunomide (10 µM), or A771726 (1 µM or 10 µM) diluted in embryo water or had their caudal fin amputated prior to treatment with the test compounds. Embryos were then incubated for 3 days followed by morphological evaluation and immunohistochemical staining.

### Zebrafish CYP1A Immunohistochemistry

Following exposure to the indicated compounds, embryos were euthanized with tricaine and fixed overnight in 4% paraformaldehyde followed by immunostaining of zfCYP1A as described previously.[9] The primary monoclonal antibody used was C107 mouse anti- zfCYP1A, 1∶500 (Biosense Laboratories, Bergen, Norway). The secondary antibody used was Alexa-546 conjugated goat anti-mouse, 1∶1000 (Molecular Probes, Eugene, OR). Embryos were mounted in 50% glycerol on a glass coverslip and imaged with an Axiovert 200 M (Zeiss).

### Lymphocyte Proliferation Assay

C57BL/6J (B6) mice and B6.129-AhRtm1Bra/J (AhR −/−) mice (purchased from The Jackson Laboratory) were bred and maintained in our specific pathogen-free animal facility at Oregon State University. All animal procedures were approved by the Institutional Animal Care and Use Committee. Spleens of AhR +/+ and AhR −/− mice were removed and whole splenocyte suspensions were prepared. Cells were then labeled with carboxyfluorescein succinimidyl ester (CFSE) [35] and cultured with 250 ng/mL anti-CD3 and 10 µg/mL LPS in the presence of 0, 5, 10 or 50 µM leflunomide or 10 or 50 µM A771726. After 72 hours, the cells were harvested and stained with fluorochrome-labeled antibodies to CD4 and CD8 and B220 (BD Pharmingen) for flow cytometric analysis. Subset-specific cell division was measured based on the two-fold dilution of CFSE fluorescence that occurs with each round of cell division. Flow analysis was performed using a Beckman Coulter FC500 flow cytometer and analyzed with WinList software (Verity Software House).

### Statistical Analysis

Reporter gene studies and leflunomide proliferation assays in AhR +/+ or AhR −/− splenocytes were analyzed by one and two-way ANOVA, respectively. Values of p<0.05 were considered statistically significant.

## Results

### Leflunomide Induces AhR-Response Element-Containing Reporter Genes

**Figure 1:**
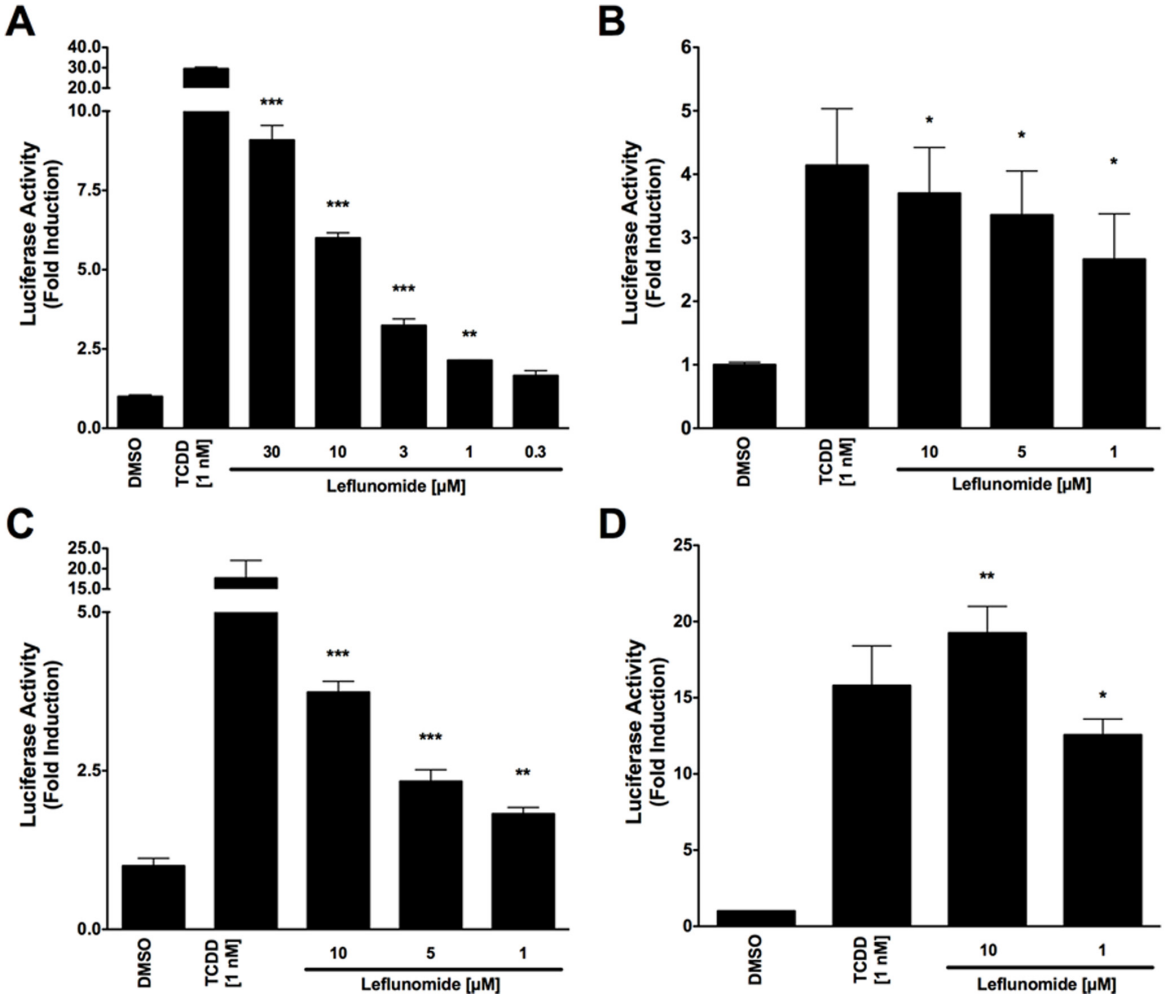
Induction of AhR-mediated reporter gene activity by leflunomide. (A) Hepa1.1 cells were treated with varying doses of leflunomide ranging from 0.3 to 30 µM for 4 hours and luciferase activity was measured. Treatment with 1 nM TCDD was included as a positive control. Leflunomide strongly induced the luciferase reporter gene starting from 1 µM. (B) WT mouse Hepa1 hepatoma or human HepG2 hepatoma cells (C) were transiently transfected with an XRE-Luc reporter gene and β-galactosidase expression vector as described in the methods section. Cells were treated with leflunomide or TCDD as indicated for 16 hours and assayed for luciferase activity; Luciferase values are normalized to β-galactosidase activity. In both WT Hepa1 and HepG2 cells, treatment with 10, 5, or 1 µM significantly increased reporter gene activity, although to a lesser extent than 1 nM TCDD. (D) HepG2 cells were co-transfected with an Arnt expression vector (300 ng) along with the XRE-Luc reporter gene (600 ng). A significant increase in the XRE reporter gene activity was observed compared to HEPG2 cells transfected with the XRE reporter construct alone. Results are the mean ± SEM of at least three independent experiments. *** p<0.0001, ** p<0.001, * p<0.05 as determined by ANOVA with a Newman-Keuls multiple comparison post-test.

Following the identification of leflunomide as a putative AhR ligand from our small molecule screening, we set out to further characterize the compound's ability to activate AhR transcription. To this end, we performed reporter gene assays to investigate the dose-dependent activation of the AhR by leflunomide. We first used a mouse hepatoma cell line stably expressing an AhR responsive luciferase reporter gene (Hepa1.1) [24] and found that leflunomide activated AhR transcription starting from 1 µM in a dose-dependent manner (Figure 1A), which was in accordance with our screening data and other published studies.[17] To further assess leflunomide activation of the AhR, we transiently transfected human HepG2 and mouse WT Hepa1 hepatoma cells with a luciferase reporter gene expression vector under control of a promoter containing xenobiotic response elements (XRE-Luc). Consistent with our observations in Hepa1.1 cells, we observed activation of AhR-mediated transcription in both Hepa1 (Figure 1B), and HepG2 (Figure 1C) cell lines. When we transiently expressed additional levels of Arnt in HepG2 cells (Figure 1D), induction of the XRE reporter increased significantly.

### Activation of AhR Target Genes by Leflunomide

**Figure 2:**
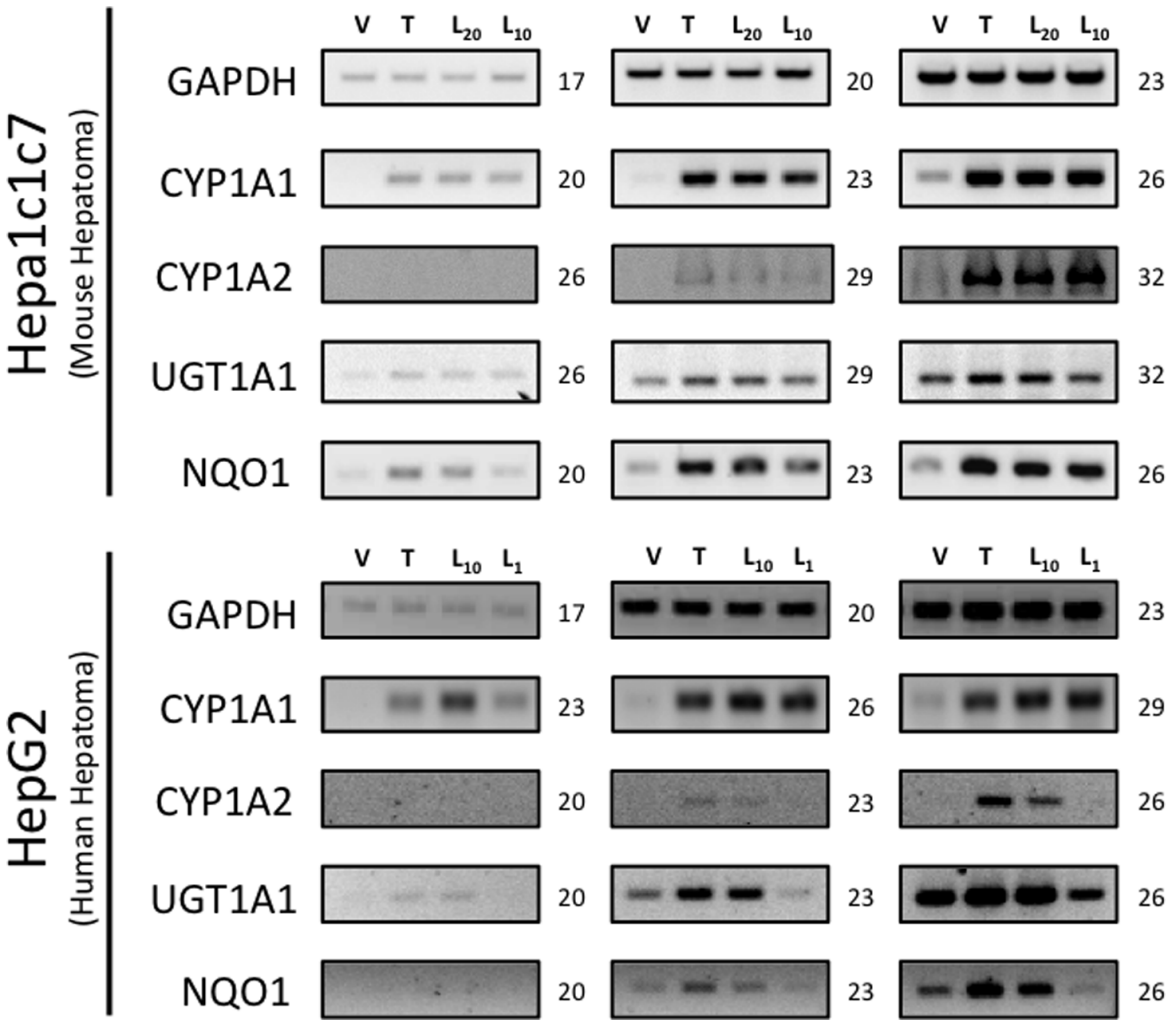
Activation of the AhR target genes by leflunomide. Hepa1 and HepG2 cells were treated with vehicle (V, 0.1% DMSO), TCDD (T, 1 nM), or leflunomide (L20, 20 µM; L10, 10 µM; L1, 1 µM) for 18 hours. RNA was isolated and semi quantitative RT-PCR was performed for the AhR target genes Cytochrome P450 1A1 (CYP1A1), Cytochrome P450 1A2 (CYP1A2), UDP glucuronosyltransferase 1A1 (UGT1A1), and NAD(P)H dehydrogenase [quinone 1] (NQO1), as described in the methods section. Expression analysis of GAPDH (Glyceraldehyde-3-phosphate dehydrogenase) was performed as a control. Cycle numbers during which PCR reactions were sampled are indicated. PCR products were visualized on a 2% TAE agarose gel stained with ethidium bromide. Leflunomide activated the selected panel of AhR target genes in WT Hepa1 (A) and HepG2 cells (B).

After confirming leflunomide's ability to induce AhR-responsive reporter genes, we next evaluated leflunomide's ability to modulate expression of several known endogenous AhR target genes by semi-quantitative PCR. First, using WT Hepa1 cells, we evaluated CYP1A1, CYP1A2, NQO1, and UGT1A1 expression with increasing concentrations of leflunomide. As shown in Figure 2A, leflunomide exposure strongly induced the transcription of CYP1A1, CYP1A2, and NQO1 similar to the maximal expression produced by 1 nM TCDD exposure. In addition, despite a significant basal expression of UGT1A1 in WT Hepa1 cells, both leflunomide and TCDD modestly upregulated UGT1A1. We also evaluated CYP1A1, CYP1A2, NQO1, and UGT1A1 expression following leflunomide exposure in HepG2 human hepatoma cells. Consistent with the results obtained from the Hepa1 cells, leflunomide activated all of the AhR target genes in a dose-dependent manner in HepG2 cells. Together, these data indicate that leflunomide increases expression of several AhR target genes involved in drug metabolism in both human and mouse cells.

### Activation of AhR Target Genes by Leflunomide Requires the AhR Heterodimerization Partner Arnt

**Figure 3:**
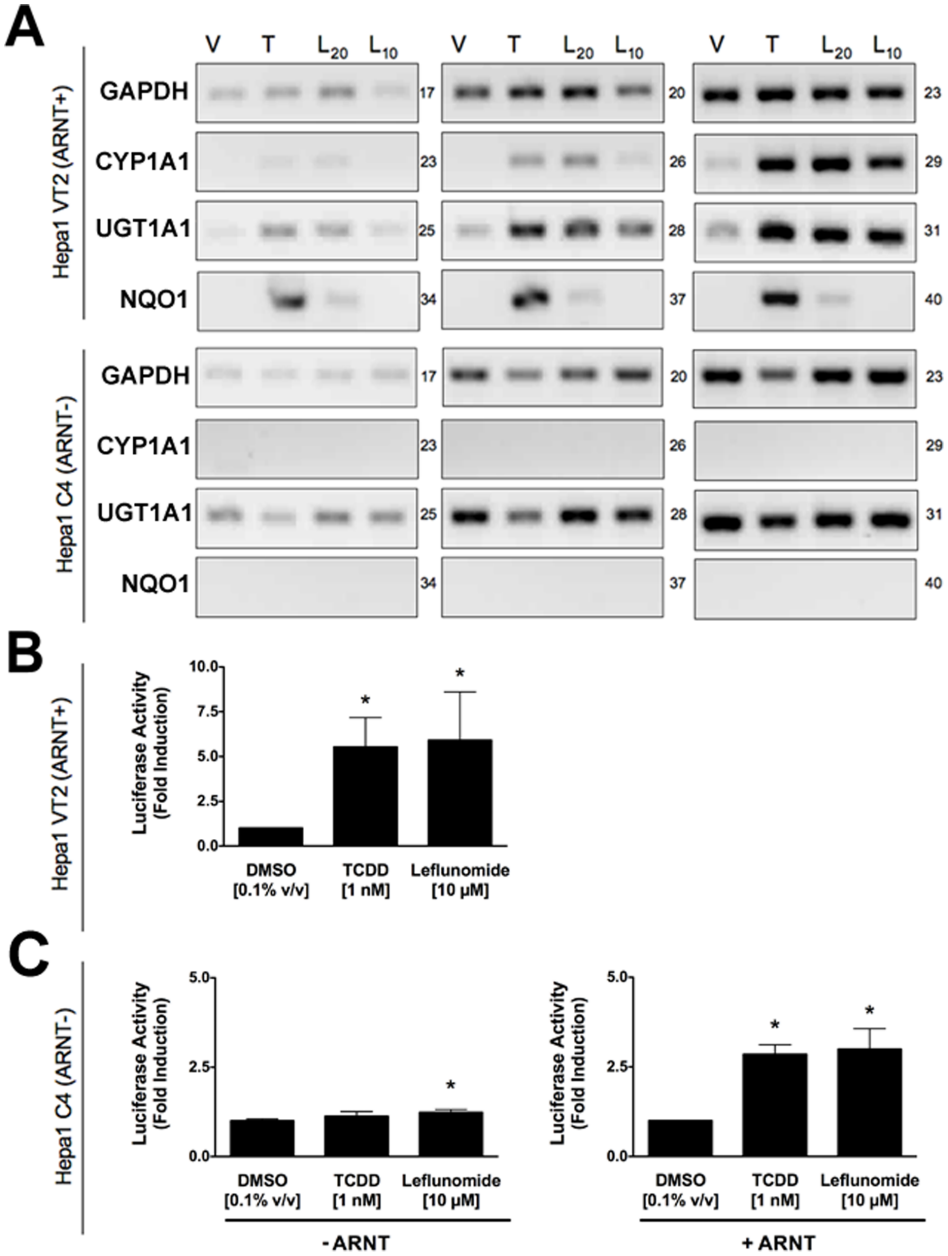
Activation of AhR target genes by leflunomide requires Arnt. (A) Induction of the AhR target genes CYP1A1, UGT1A1, and NQO1 was performed as in figure 2. Vehicle (V, 0.1% DMSO), TCDD (T, 1 nM), leflunomide (L20, 20 µM; L10, 10 µM). GAPDH expression was used as a control. PCR cycle numbers are indicated. Target gene induction in Hepa1 vT{2} cells expressing a functional Arnt protein was similar as to that seen with WT Hepa1 cells. CYP1A1 and NQO1 were not induced by leflunomide in Hepa1 C4 cells that do not express a functional Arnt protein. (B–C) XRE-Luc reporter gene assays in Hepa1 vT{2} and C4 cells. Cells were transfected and treated with leflunomide or controls (VEH, Vehicle, 0.1% v/v DMSO; TCDD, 1 nM; and LEF, leflunomide,10 µM) as described in the methods section. (B) Consistent with semi quantitative RT-PCR analysis, TCDD and leflunomide induced expression of the XRE-Luc reporter gene in Hepa1 vT{2} cells. (C) Treatment with TCDD or leflunomide failed to activate the XRE-Luc reporter in the Hepa1 C4 cells. However, transient co-expression of Arnt rescued XRE-Luc reporter gene induction. Reporter gene assays are the mean ± SEM of three independent experiments.

Given that the structure of leflunomide differs significantly from that of the classical ligand, TCDD, we wanted to confirm that its ability to activate transcription of AhR target genes was dependent on the presence of a functional Arnt protein, which would indicate that leflunomide activates AhR signaling through the classical pathway. To this end, we used the C4 mutant cell line, which has non-functional Arnt activity (Arnt G326D) [25], and Hepa1 VT{2} cells, which have restored Arnt activity due to stable re-expression of wildtype Arnt.[23] We performed semi-quantitative PCR to evaluate target gene induction in the two Hepa1 derivative cell lines following treatment with leflunomide (Figure 3A). The AhR target genes CYP1A1, UGT1A1, and NQO1 were induced in the Arnt transcription proficient cell line following exposure to leflunomide; however, induction of NQO1 was considerably weaker. Neither CYP1A1 nor NQO1 was induced by leflunomide in the Hepa1 C4 cell line, whereas basal UGT1A1 expression was not altered. Furthermore, leflunomide activated XRE-Luc reporter gene activity in Hepa1 vT{2} cells in a dose-dependent manner, with a maximal induction comparable to that of TCDD (Figure 3B), while neither TCDD nor leflunomide increased reporter gene activity in the Arnt deficient Hepa1 C4 line (Figure 3C). To verify the results observed in vT{2} cells, C4 cells were also transiently co-transfected with an expression vector for Arnt, which rescued induction of the XRE-Luc reporter by both TCDD and leflunomide (Figure 3C).

### Leflunomide, but not its Biologically Active Metabolite, A771726, Activates the AhR

**Figure 4:**
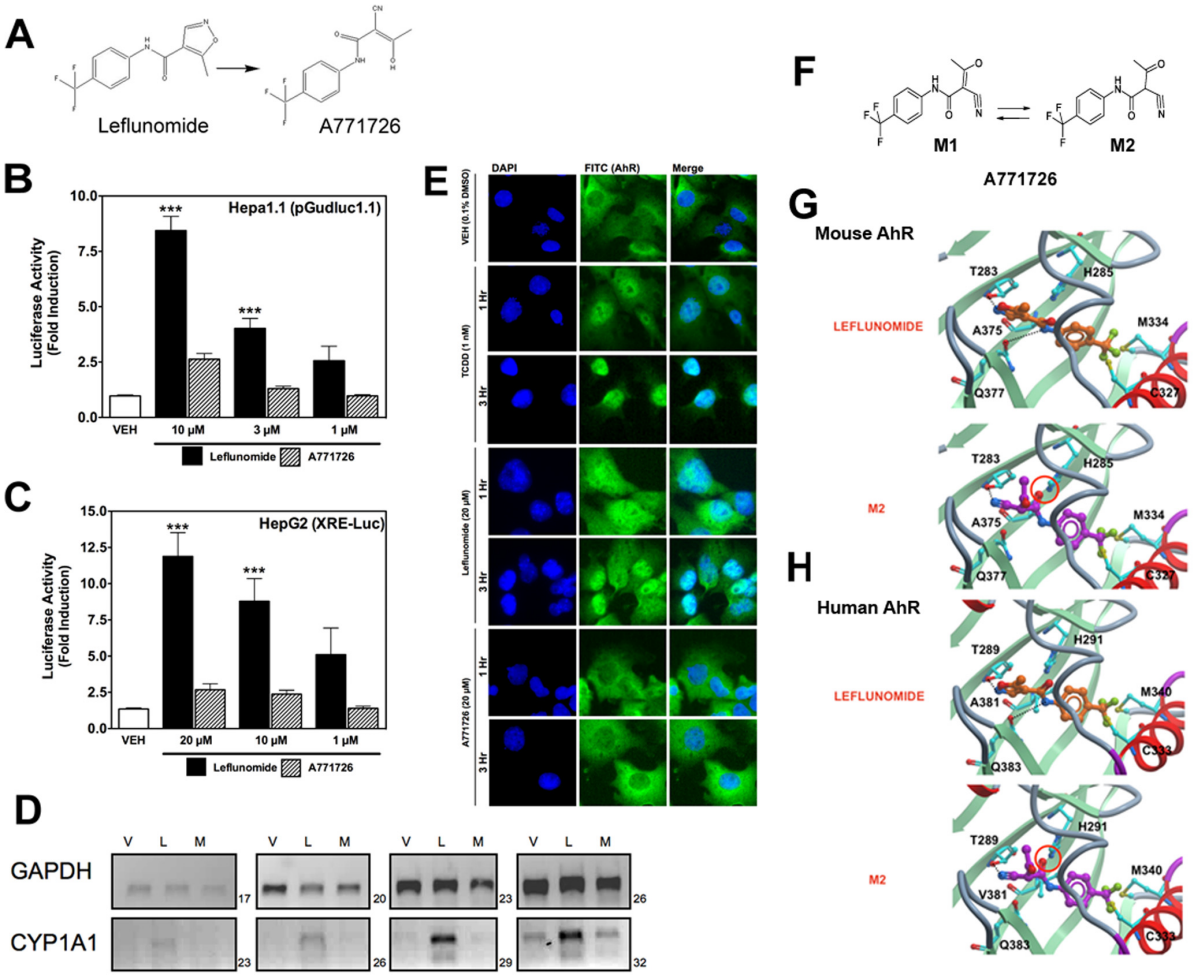
Leflunomide, but not its active metabolite, A771726, activates the AhR. (A) Structures of leflunomide (left) and its metabolite A771726 (right). (B–C) Reporter gene assays were conducted in Hepa1.1 cells or in HepG2 cells transiently transfected with XRE-Luc reporter gene. Results are the mean ± SEM of at least three independent experiments, each of which consisted of at least three biological replicates. ***: p<0.001 compared with vehicle treatment and p<0.05 compared with corresponding dose of A771726. (D) To confirm the observations of the reporter gene assays, we performed semi quantitative RT-PCR in WT Hepa1 cells for CYP1A1 with total RNA isolated from cells treated with vehicle (0.1% DMSO), leflunomide (L, 20 µM) or A771726 (M, 20 µM) as described in Figure 2. GAPDH was included as a control. Consistent with reporter gene assays, A771726 failed to activate CYP1A1 beyond that of vehicle treatment, while leflunomide induced strong CYP1A1 expression. (E) Cellular localization of AhR was analyzed by immunofluorescence of Hepa1 cells treated with TCDD, leflunomide, or A771726 for 1 or 3 hours. The FITC (green) channel represents AhR staining, while DAPI (blue) represents the nucleus. The AhR translocated to the nucleus following treatment with both TCDD and leflunomide, while it remained in the cytosol following treatment with A771726. (F) M2 is the major tautomeric form of A771726. Molecular docking of M2 and leflunomide in the homology models of mouse (G) and human (H) AhR ligand binding domain reveal favorable energetic and docking for leflunomide but not M2.

Previously, an *in vitro* metabolism study on isoxazole ring scission found that leflunomide may be metabolized via a cytochrome P450-mediated isoxazole ring opening N-O bond cleavage to form its active metabolite, A771726.[21] Given the significant structural change incurred during the metabolism of leflunomide to A777176 (Figure 4A), we were interested to determine whether the metabolite could also activate the AhR. To this end, we performed reporter gene assay with both leflunomide and A771726 in Hepa1.1 cells (Figure 4B) and HepG2 cells (Figure 4C). While leflunomide activated the reporter gene in a concentration-dependent manner, A771726 failed to significantly induce the AhR-dependent reporter gene in both the cell lines. We also evaluated the induction of CYP1A1 by leflunomide and A771726 using semi-quantitative RT-PCR (Figure 4D). Consistent with the reporter gene data, A771726 failed to induce CYP1A1 expression beyond that of vehicle treatment, while leflunomide strongly induced CYP1A1 expression, consistent with earlier observations.

To further confirm our observation that A771726 is not an AhR agonist, we evaluated the ability of both leflunomide and A771726 to facilitate AhR translocation from the cytosol to the nucleus (Figure 4E). After treatment for 1 and 3 hours, both TCDD and leflunomide strongly promoted AhR nuclear localization, while A771726 did not. Together, these data indicate that leflunomide but not its metabolite, A771726, is a ligand of the AhR.

### Molecular Docking of Leflunomide and A771726

We were next interested in determining if there is a structural explanation for the observed disparity between the AhR activation by leflunomide, but not its metabolite. To this end, leflunomide and its metabolite A771726 (Figure 4A) were docked into the mouse and human AhR-LBD homology model as previously described. [33] For the primary metabolite, A771726, the most predominant tautomeric form in solution, M2 [21], was considered in the study (Figure 4F). Both compounds docked into the binding pocket, but produced distinct binding energetics. Specifically, leflunomide established two hydrogen bond (HB) interactions between the nitrogen atom of the isoxazole ring of the ligand and the OH of the side chain of Thr 289/283 and between the amide NH of the ligand and the carbonyl CO of the side chain of Gln 383/377 (Figure 4G) with a predicted binding energy of −2.65 kcal/mol (human) and −2.84 kcal/mol (mouse). Conversely, A771726 established only a single HB interaction between the nitrogen of the CN of the compound and the OH of the side chain of Thr 289/283 (Figure 4H) but with a high unfavorable (+77.5 kcal/mol (human); +40.48 kcal/mol (mouse)) binding energy. This was due to the clash in the binding pocket between the carbonyl oxygen CO of the amide group of the compound and the side chain of His 291/285 (Figure 4G). Thus, the observed AhR agonistic activity of leflunomide as well as inactivity of A771726 could be explained by *in silico* molecular docking.

### Leflunomide, but not its Active Metabolite, A771726, Induces AhR-dependent CYP1A Expression *in vivo*

**Figure 5:**
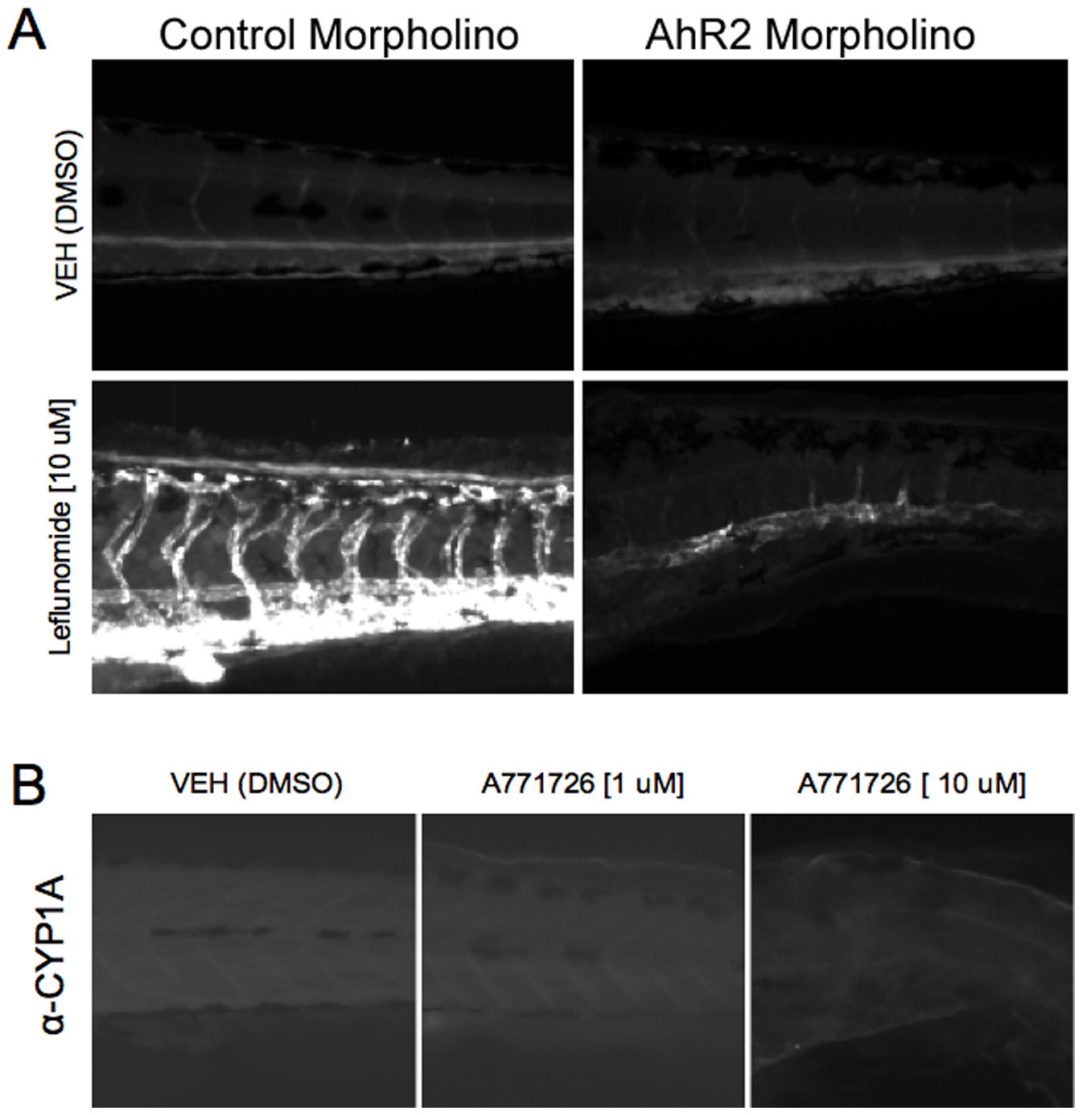
Leflunomide induces aryl hydrocarbon receptor dependent expression of CYP1A1 in zebrafish. (A) One-cell stage wildtype embryos were injected with a control morpholino or AhR2 morpholino. At 6 hpf, the zebrafish were exposed to 10 µM leflunomide for 3 days. Immunohistochemistry for CYP1A, a known AhR2 target gene in zebrafish, demonstrated that leflunomide induces CYP1A expression in an AhR2 dependent manner. (B) Exposure of zebrafish to A771726 at doses of either 1 or 10 µM did not increase CYP1A expression.

Following characterization of AhR activation by leflunomide in mouse and human hepatoma cells, we evaluated the functional consequences of AhR activation *in vivo* using the embryonic zebrafish model.[9] We first evaluated whether leflunomide exposure induced AhR target gene CYP1A *in vivo*. Zebrafish embryos were waterborne exposed to 10 µM leflunomide from 6 hpf to 120 hpf, after which CYP1A expression was evaluated by immunohistochemistry. In the absence of exogenous ligand, CYP1A expression was undetectable, and leflunomide exposure strongly induced CYP1A expression throughout the larval vasculature. Zebrafish have three AhR isoforms and AhR2 was shown to be the dominant isoform that regulates CYP1A in response to TCDD treatment.[8] To determine if the leflunomide-mediated CYP1A induction was AhR2-dependent, we used AhR2 morpholinos to repress AhR2 expression. While leflunomide induced strong CYP1A expression in control morphants (Figure 5A), CYP1A expression was markedly reduced in the AhR2 morphants, indicating that the induction was AhR2-dependent. However, exposure to A771726 did not increase CYP1A expression (Figure 5B). Taken together, these data indicate that leflunomide, but not its metabolite, activates the AhR *in vivo*.

### Leflunomide Blocks AhR2-Dependent Fin Regeneration

**Figure 6:**
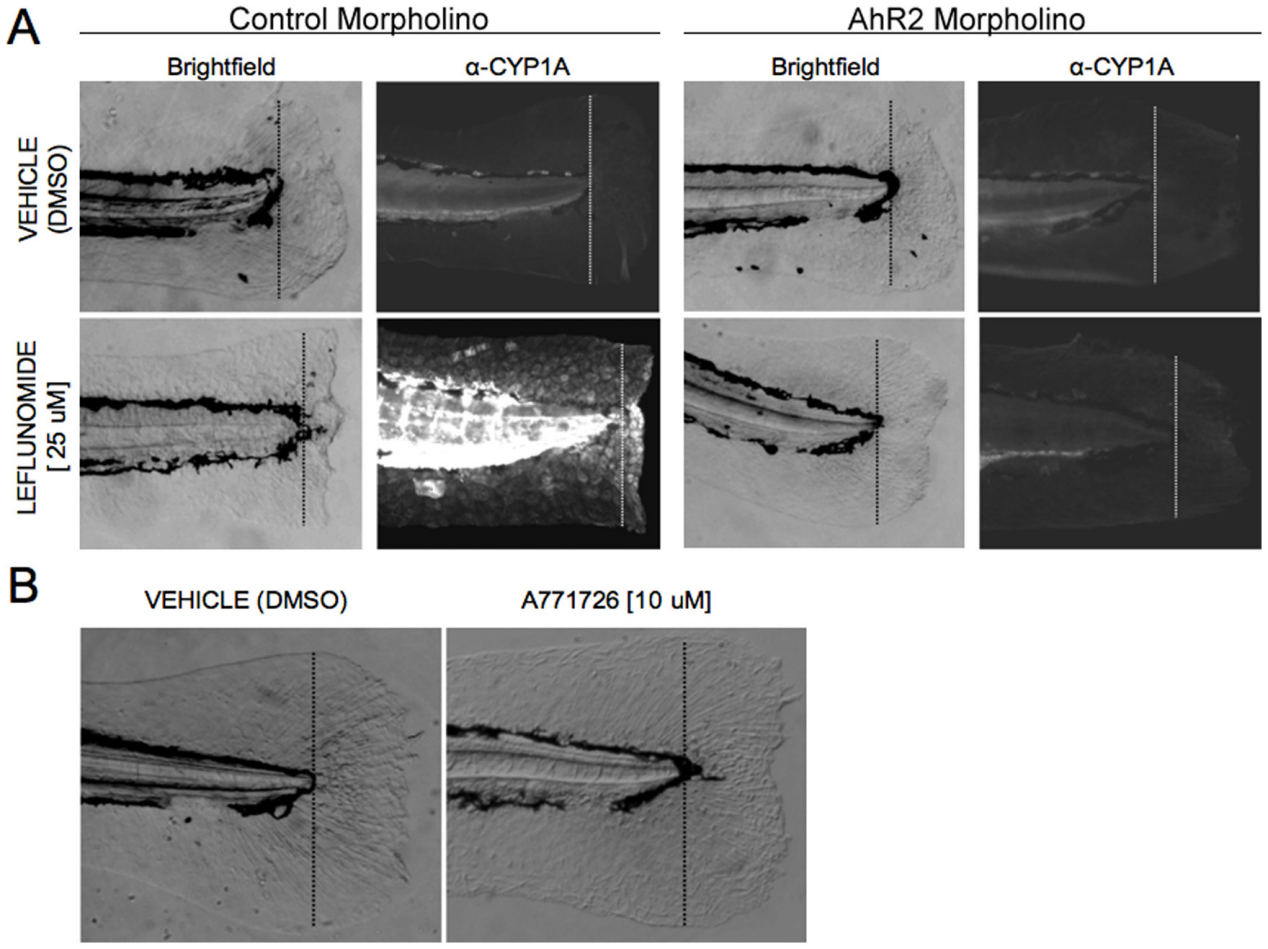
Leflunomide acts through the AhR to inhibit regeneration in an AhR-dependent manner. Amputation of the caudal zebrafish fin is a well-established model used to study tissue regeneration; TCDD is known to inhibit this regeneration process. To investigate whether leflunomide behaves like TCDD to inhibit epimorphic regeneration through the AhR, one-cell stage embryos were injected with a control or AhR2 morpholino, and the caudal fin was amputated at 48 hpf, followed by immediate exposure to vehicle or 25 µM leflunomide. (A) After 3 days, images of the fins were taken with brightfield microscopy. In addition, immunohistochemistry for CYP1A confirmed that leflunomide activated AhR2 (dotted line indicates the plane of amputation). (B) Although A771726 did not appear to activate the AhR *in vivo* or in our cell culture models, we investigated whether the compound could also inhibit fin regeneration, as inhibition of cell growth via disruption of *de novo* pyrimidine biosynthesis inhibition is a well-characterized endpoint of A771726. Amputated zebrafish were exposed to vehicle or 10 µM A771726. Exposure to 10 µM A771726 did not influence the regenerative process.

Activation of the AhR modulates cell differentiation and proliferation in a variety of contexts.[3], [4], [36] We previously developed zebrafish fin regeneration models to identify the mechanism by which AhR alters signal transduction pathways *in vivo*. Inappropriate activation of the AhR by TCDD completely abrogates tissue regeneration[8] via an AhR2-dependent mechanism.[9] AhR blocks the regenerative response by cross-talk with the β-catenin and Wnt signaling pathways.[37] To determine if leflunomide exposure would block fin regeneration via AhR2 activation, we generated AhR2 morphants, amputated the caudal fins at 48 hpf, and immediately exposed them to vehicle or leflunomide for 3 days, after which the extent of tissue regeneration was assessed by standard light microcopy. Leflunomide exposure completely blocked fin regeneration in the control morphants, but regeneration was restored in the AhR2 morphants (Figure 6A). Immunohistochemistry revealed that CYP1A was highly expressed in the leflunomide-exposed control morphants and CYP1A expression was significantly reduced in leflunomide-exposed AhR2 morphants (Figure 6A). These results demonstrate that leflunomide behaves as an AhR ligand *in vivo*. In contrast, A771726 was unable to activate the AhR and did not impact fin regeneration (Figure 6B). Since A771726 did not block regeneration, this suggests that the regenerative process is not responsive to dihydroorotate dehydrogenase inhibition, suggesting that the observed effects of leflunomide in zebrafish are independent of its metabolite, A771726.

### The AhR is not required for the anti-proliferative effects of leflunomide in primary T cells

**Figure 7:**
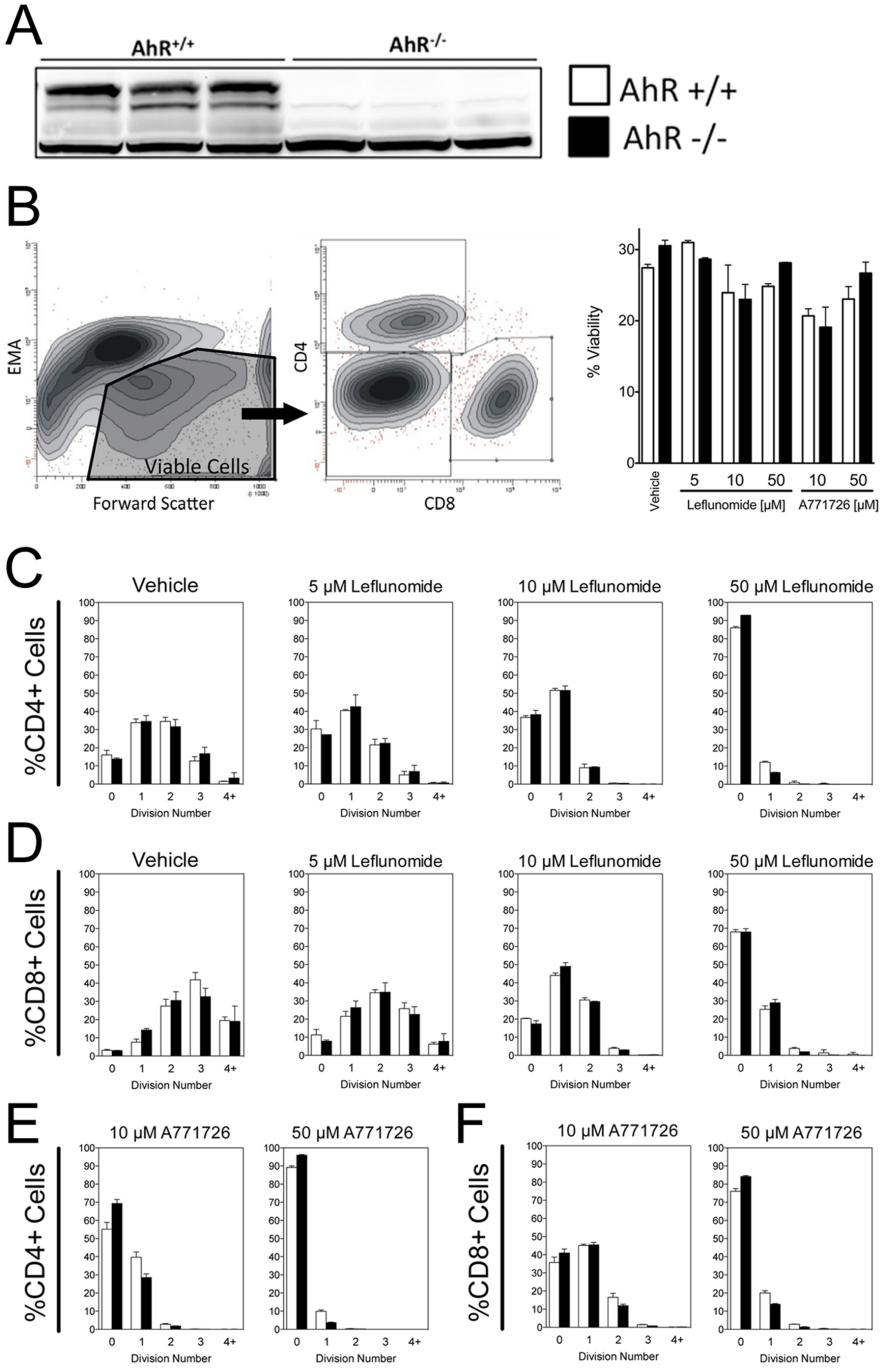
The AhR is not required for the suppression of murine T-cell proliferation by leflunomide. Spleens of AhR +/+ and AhR −/− mice (A, Western blot) were removed and whole murine splenocytes were labeled with Carboxy-Fluoroscein-Diacetate Succinimidyl-Ester (CFSE) and stimulated to proliferate *in vitro* with anti-CD3 and LPS treatment in the absence or presence of vehicle and different doses of leflunomide or A771726. After 3 days, cells were harvested and stained for CD4 or CD8. (B) Viable cells were gated and CD4 and CD8 positive cell populations were then analyzed using single parameter (CFSE) histograms to evaluate cellular divisions based on CFSE staining. Cellular viability was not affected by either leflunomide or A771726 at the concentrations used. (C) Dose-dependent inhibition of cellular proliferation by leflunomide in CD4^+^ cells is not dependent upon the AhR. (D) Dose-dependent inhibition of cellular proliferation by leflunomide in CD8^+^ cells is not dependent upon the AhR. (E–F) A771726-induced inhibition of CD4^+^ or CD8^+^ proliferation is similar between AhR +/+ and AhR −/− cells. AhR −/− cells, black bars; AhR +/+ cells, white bars. Results are the mean from two independent experiments. Two-way ANOVA for both CD4^+^ and CD8^+^ assays did not reveal any statistically significant differences between cell divisions in AhR +/+ and AhR −/− cells whereas the effect of leflunomide on cell division compared to vehicle treatment was highly significant in both cases (p<0.0001).

Given the observation that leflunomide was able to elicit an AhR-dependent inhibition of regeneration in the zebrafish model, we next asked whether AhR is involved in the immunosuppressive action of leflunomide or A771726 in CD4^+^ or CD8^+^ T cells from AhR −/− and AhR +/+ backgrounds (Figure 7A). In order to evaluate the effects of leflunomide and A771726 on cell proliferation, we confirmed that leflunomide or A771726 did not alter the viability of the analyzed cell populations compared with vehicle treatment (Figure 7B). Viable CD4^+^ or CD8^+^ T cells (Figure 7B) from AhR +/+ or AhR −/− mice were then analyzed as a function of CFSE staining to determine the number of cellular divisions that had taken place. The growth inhibitory actions of both leflunomide and A771726 in CD4^+^ and CD8^+^ T-cells were readily apparent when compared to vehicle treated cells. Leflunomide potently reduced the number of cell divisions in both CD4^+^ and CD8^+^ T-cells (Figures 7C–D). Specifically, leflunomide at 5 µM suppressed the number of cells undergoing three or more divisions, whereas leflunomide at 50 µM suppressed proliferation beyond one division. (Figures 7C–D). However, no difference was observed in the growth inhibition patterns by leflunomide in cells from AhR positive or null mice (Figures 7C–D). A771726 was also able to inhibit proliferation of CD4^+^ or CD8^+^ T cells, but no difference was observed between AhR +/+ and AhR −/− cells. In addition, the degree of inhibition by A771726 was similar to that of leflunomide Figures 7E–F). Together, these data suggest that neither the metabolism of leflunomide to A771726 nor their respective anti-proliferative effects could be linked to AhR activation in mouse splenocytes.

### Inhibition of CYP1A2 increases leflunomide-induced AhR reporter gene activity

**Figure 8:**
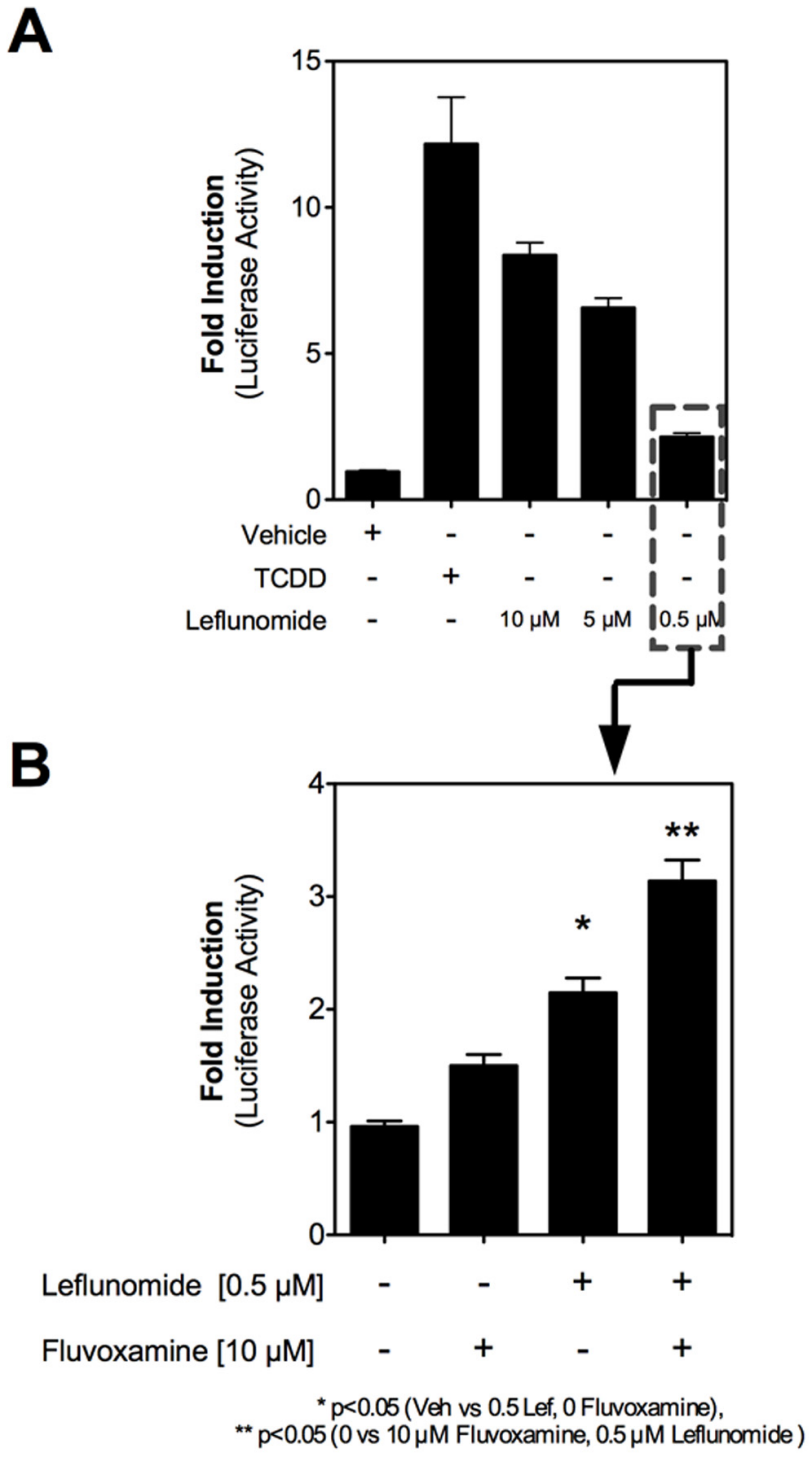
Inhibition of CYP1A2 by fluvoxamine increases AhR-mediated reporter gene activity. A) Identification of limiting doses of leflunomide capable of activating the AhR. Hepa1.1 cells were treated for 4 hours with varying doses of leflunomide in order to identify concentrations necessary for minimal activation of reporter gene activity. Leflunomide at 0.5 µM (dashed box) resulted in an approximately 2-fold induction of reporter gene and was selected for further analysis with the CYP1A2 inhibitor fluvoxamine. B) The CYP1A2 inhibitor fluvoxamine increases leflunomide-induced reporter gene activity in Hepa1.1 cells. Hepa1.1 cells were pre-treated with fluvoxamine for 3 hours and then dosed with 0.5 µM leflunomide for an additional 4 hours. Leflunomide-mediated reporter gene activity was enhanced by pre-treatment with fluvoxamine compared with vehicle treated cells and fluvoxamine-only treated cells. Results are the mean ± SEM of three independent experiments, each with at least three biological replicates. * p<0.05 relative to vehicle treatment, ** p<0.001 relative to both vehicle treatment and vehicle treated cells pre-treated with fluvoxamine.

As shown in Figure 4, cytochrome P450-mediated isoxazole ring scission leading to the conversion of leflunomide to A771726 results in a loss of ability to activate the AhR. To further study the metabolism of leflunomide to A771726, and specifically the role of CYP1A2 (and to a lesser extent CYP1A1, due to its genetic similarity to CYP1A2) in this metabolism, we utilized fluvoxamine, a chemical inhibitor of CYP1A2 (as well as CYP1A1). [38] First, we identified the lowest dose of leflunomide that can activate AhR in Hepa1.1 cells (Figure 8A). Next, we pre-treated the cells with fluvoxamine before treating them with a limiting leflunomide concentration and assessed luciferase activity. As would be expected for a role for CYP1A1/2 in the metabolism of leflunomide to A771726,[21] we observed a modest, but nevertheless statistically significant increase in AhR-mediated luciferase reporter gene activity upon chemical inhibition of CYP1A1/2 (Figure 8B).

## Discussion

The AhR is gaining interest as a potential therapeutic target for the treatment of immune-mediated diseases.[12], [15], [39] While the AhR is commonly associated with TCDD toxicity, it is increasingly clear that there are aspects of AhR activation that can be beneficial. Activation of AhR activity by TCDD has been shown to suppress autoimmune disease development, including that of type 1 diabetes and Experimental Autoimmune Encephalomyelitis.[15], [40], [41], [42], [43], [44] However, clinical use of TCDD is unlikely due to its history as an environmental toxicant. Therefore, we searched for AhR ligands that trigger the beneficial aspects of the AhR without unwanted side-effects such as those seen with TCDD. Pharmaceuticals already in use in the clinic are ideal candidates to screen for such selective AhR activation, as many potential toxicity issues have already been addressed. In the present study, we coupled the identification of hits from a small molecule screen for novel AhR ligands with a rapid *in vivo* model for testing the consequences of AhR activation.[9]

Mimicking the beneficial effects of TCDD (i.e. immunosuppression) with alternative non-toxic AhR ligands may prove to be a useful therapeutic strategy for immune-mediated diseases. In our search for alternative AhR ligands, we found the anti-inflammatory drug, leflunomide, activated the AhR, resulting in induction of several known AhR target genes. Interestingly, we also observed that leflunomide induced CYP1A2 expression. Further, consistent with the observation that CYP1A2 can facilitate metabolism of leflunomide to A771726 *in vitro*,[21] we found that chemical inhibition of CYP1A2 enhanced leflunomide-induced AhR reporter gene activity. Induction of drug metabolizing enzymes (DMEs), following exposure to xenobiotics has been established as a major activity of the AhR.[1] In light of this fact, our results suggest a possible role for the AhR in mediating the conversion of leflunomide to A771726. However, other DMEs have also been shown to facilitate conversion of leflunomide to A771726.[21], [45]

Identification of novel AhR ligands and subsequent analysis of their effects on AhR biology is important for developing the AhR as a clinically relevant therapeutic target. Indeed, given that leflunomide can activate the AhR *in vivo* and produce an AhR-dependent effect on tissue regeneration, future studies may reveal useful therapeutic applications of leflunomide or related compounds. The AhR's ability to regulate cell proliferation may also be exploited for therapeutic purposes including cancer treatment.

In conclusion, we found that leflunomide, but not its metabolite A771726, is an agonist of the AhR. Leflunomide was able to activate the AhR *in vivo*, where it was able to mediate AhR-dependent effects on tissue regeneration. As leflunomide is a drug currently in use in the clinic and is an agonist of the AhR, our results support the feasibility of developing AhR-targeted therapeutics.
